# “You’ve got a friend in me”: can social networks mediate the relationship between mood and MCI?

**DOI:** 10.1186/s12877-017-0542-0

**Published:** 2017-07-13

**Authors:** Jennifer A. Yates, Linda Clare, Robert T. Woods, The Cognitive Function and Ageing Study: Wales

**Affiliations:** 1 Institute of Mental Health, University of Nottingham, Nottingham, UK; 2 Department of Psychology and PenCLAHRC, University of Exeter, Exeter, UK; 3 Dementia Services Development Centre, Bangor University, Bangor, UK

**Keywords:** Mild cognitive impairment, Anxiety, Depression, Social networks, Cognition

## Abstract

**Background:**

Social networks can change with age, for reasons that are adaptive or unwanted. Social engagement is beneficial to both mental health and cognition, and represents a potentially modifiable factor. Consequently this study explored this association and assessed whether the relationship between mild cognitive impairment (MCI) and mood problems was mediated by social networks.

**Methods:**

This study includes an analysis of data from the Cognitive Function and Ageing Study Wales (CFAS Wales). CFAS Wales Phase 1 data were collected from 2010 to 2013 by conducting structured interviews with older people aged over 65 years of age living in urban and rural areas of Wales, and included questions that assessed cognitive functioning, mood, and social networks. Regression analyses were used to investigate the associations between individual variables and the mediating role of social networks.

**Results:**

Having richer social networks was beneficial to both mood and cognition. Participants in the MCI category had weaker social networks than participants without cognitive impairment, whereas stronger social networks were associated with a decrease in the odds of experiencing mood problems, suggesting that they may offer a protective effect against anxiety and depression. Regression analyses revealed that social networks are a significant mediator of the relationship between MCI and mood problems.

**Conclusions:**

These findings are important, as mood problems are a risk factor for progression from MCI to dementia, so interventions that increase and strengthen social networks may have beneficial effects on slowing the progression of cognitive decline.

## Background

Mood problems occur frequently in older people with mild cognitive impairment (MCI) and may represent a risk for greater progression from MCI to dementia [1–3]. Therefore, it is important to explore factors that may contribute to the development of mood problems in people with MCI. A better understanding of the complicated relationship between MCI and low mood, through investigating the influence of psychosocial and environmental factors, such as social networks, may assist in the development of practical interventions that may help to slow or prevent cognitive decline.

Social networks can be conceptualised as the web of social relationships surrounding a person, taking into account the nature of the interpersonal ties involved [4]. Whilst most older people do have a significant number of relationships, there is a negative association between age and social network size [5] as older people experience a reduction in the size of their social networks, and are more likely to disengage socially than younger people [6]. This may be due to the death of friends and family members, problems getting out of the house, a loss of confidence, or a lack of opportunities for social interaction. Older people may also perform a selective ‘pruning’ process where contact is lost with peripheral members of social networks in order to focus on more meaningful relationships with emotionally closer members of the network [7].

There is a relationship between social networks and mood problems in general, as people with fewer social interactions are more likely to report symptoms of anxiety or depression [8], and having multiple social roles can help to promote self-esteem, which in turn may prevent depression [4, 9]. Some studies have indicated that people who experience greater levels of social interaction and support have better mental health outcomes [10, 11] and significantly decreased psychological distress [6]. Social cognitive theory [12] suggests that experiencing anxiety or depression may reduce feelings of efficacy necessary to create and maintain relationships, leading to a decline in social networks. However, it is not clear at this stage if the relationship between social networks and mood operates in the same way for people with MCI.

There is also evidence for a link between cognitive function and social networks [4, 13]. Social isolation or disengagement is thought to accelerate cognitive decline in ageing [9, 14] whereas a rich social network is thought to have a protective effect against dementia [14]. Cognitive impairment may make it difficult for older people to maintain social networks, leading to a withdrawal from social interactions and in turn an increase in anxious or depressive symptomatology, although current research remains divided on this with some studies supporting the idea [15] and others opposing it [16]. On the other hand, increased symptoms of anxiety or depression may lead to fewer social interactions, increasing the risk of developing cognitive impairment.

Consequently, this area of research is important because it focuses on a modifiable factor that, if improved, could benefit both cognitive functioning and mood in older people, and contribute to a better quality of life for older people and their families and friends. The present study adds to the literature by investigating this area through the use of mediation analyses with a large sample size and clearly operationalised MCI criteria, which has not been seen in previous research. This study will examine the relationship between MCI, mood and social networks by addressing the following aims:

1. To examine associations between MCI, mood and social networks.
2. To investigate whether social networks account for the variance in the relationship between MCI and mood.

## Methods

### Design

Mood, cognitive functioning and social networks were examined using cross-sectional data from a large sample of older people who participated in the Cognitive Functioning and Ageing Study Wales (CFAS Wales) between 2010 and 2013. Fuller details can be found on the CFAS Wales website (http://www.cfas.ac.uk/cfas-wales/). CFAS Wales is a longitudinal population-based study which has gathered information about participants drawn from two research centres in urban and rural areas of Wales, investigating changes that people may experience as they age. Participants took part in face-to-face, structured interviews, usually conducted in their own homes, with trained interviewers through the medium of English or Welsh, depending on the participant’s preference. The interviews included the Geriatric Mental State Schedule [17] which was used to diagnose dementia, mood problems and other mental health problems; the Cambridge Cognitive Examination (CAMCOG) [18] which was used to test cognitive functioning; and a variety of other health and lifestyle questions. Ethical approval was granted by the North Wales Research Ethics Committee (West). All participants provided informed consent to participate in the study, or if unable to complete the consent process personally, an appropriate consultee provided consent on the participant’s behalf. This analysis presents cross-sectional data from the first wave of interviews.

### Participants

All individuals aged over 65 years and living in the Gwynedd, Anglesey and Neath Port Talbot areas of Wales were randomly sampled between 2011 and 2013 from GP practice lists, resulting in a total sample of 3593 participants. Participants were excluded from participating in CFAS Wales if their GP felt that it would be inappropriate, for example if they were terminally ill.

The present analysis is concerned with participants who are not cognitively impaired, and those that meet criteria for MCI. Consequently, participants were excluded from the analysis if they had a diagnosis of dementia (*n* = 129), impaired activities of daily living (ADLs; *n* = 52), or cognitive decline that did not meet criteria for MCI (outlined below) but also did not meet the criteria for dementia (other cognitive impairment no dementia; OCIND; *n* = 481). Forty participants were excluded due to incomplete data, and 78 were unable to complete an interview resulting in 2813 participants included in this analysis.

### Classification of mild cognitive impairment

MCI was defined using criteria similar to Petersen et al. [19] as an objective cognitive impairment, intact ADLs, intact general cognition (indicated by a score equal to or greater than 22 on the MMSE), an absence of dementia as indicated by a case level of organicity using the AGECAT algorithm [20], and the presence of a subjective memory complaint indicated by a positive response to the question: “Have you ever had any difficulty with your memory?” which was asked during the interview. Objective cognitive impairment was defined as scores falling one standard deviation below age-adjusted norms on one or more of the subscales of the CAMCOG [21], a well-established scale designed to asses different domains of cognitive functioning that formed a section of the CFAS Wales interview.

### Social network score

Social network was assessed in the CFAS Wales interview by ascertaining participants’ frequency of contact with friends and relatives using the Lubben Social Network Scale six-item version (LSNS-6) [22]. The LSNS-6 comprises two subscales investigating the number of contacts, number of confidants and availability of support amongst family and friends, and has a maximum score of 30.

### Assessment of mood

Anxiety and depression were assessed in the CFAS Wales interview using the AGECAT algorithm [20], where a score of three or above indicated a case level of anxiety or depression. This study uses a cut off of 3 on the AGECAT and considers all participants with a score of three or above as having anxiety or depression.

### Number of health conditions

The number of health conditions was used as a covariate in multiple regression analyses. This was assessed by asking participants to indicate whether or not they had been diagnosed with any of the following health conditions: angina, intermittent claudication, high/low blood pressure, cancer, diabetes, Parkinson’s disease, stroke, heart attack, fits/epilepsy, serious head injury, chronic bronchitis, asthma (excluding childhood asthma), arthritis, peptic ulcers, pernicious anaemia, transient ischemic attack, thyroid problems, meningitis, and shingles. These questions were mapped onto the Charlson Comorbidity Index [23]. In line with the scoring system of the Charlson Co-Morbidity Index, a summary score was created by scoring each positive answer as one, except for cancer which was scored two or three depending on whether the cancer was a past or current problem. The total score had a range from 0 to 21, with higher scores reflecting higher levels of illness.

### Statistical analyses

Statistical analyses were conducted using SPSS 21.0. Regression analyses were used to investigate the change in odds of experiencing symptoms of anxiety and depression dependent on cognitive status and social network score, controlling for both age and gender, and whether social network score mediated the relationship between cognitive status and mood. Sobel’s test was used to confirm whether the mediation effect was significant.

## Results

Data were analysed from 2813 participants who were classified as having no cognitive impairment (NCI) or MCI, and their characteristics are shown in Table 1. A t-test did not reveal differences between the participants in the NCI and MCI categories in age or years in full time education and a chi-squared test did not reveal differences in marital status. Significantly more participants in the MCI category were female (Table 1).

**Table 1:** Characteristics of the study sample

		NCI	MCI	
Age mean (SD)		74.20 (6.84)	73.88 (6.12)	*t* (238.66) = 0.70, *p* = .317(equal variances not assumed)^a^
Female N (%)		1423 (54.5)	89 (44.5)	*t* (2811) = −2.73, *p* = .006
Years in full time education mean (SD)		11.85 (2.80)	11.44 (2.40)	*t* (2811) = 0.64, *p* = .526
Marital status N (%)	Married	1652 (63.2)	132 (66.0)	*χ* ^*2*^ (4) = 5.22, *p* = .265
	Cohabiting	41 (1.6)	2 (1.0)	
	Single	92 (3.5)	6 (3.0)	
	Widowed	649 (24.8)	40 (20.0)	
	Divorced/Separated	178 (6.8)	20 (10.0)	
LSNS6 score mean (SD)		15.66 (5.76)	14.15 (6.13)	
Anxiety N (%)		131 (5.0)	18 (9.0)	
Depression N (%)		617 (23.6)	78 (39.0)	
Subjective memory complaint N (%)		843 (32.3)	200 (100)	
Number of health conditions (SD)		2.96 (2.00)	3.33 (2.27)	*t* (223.24) = −2.18, *p* = .030(equal variances not assumed)^a^
Total		2613	200	

^a^Levene’s test indicated that homogeneity of variance could not be assumed between the MCI and NCI categories for this analysis and consequently a reduced number of degrees of freedom were used to increase the robustness of this test

### The association between MCI and mood

Logistic regression analyses showed the odds of having symptoms of anxiety were significantly greater in people categorised as MCI (OR = 2.06, CI = 1.10-3.86, *p* = .024), compared with those categorised NCI. The odds of having symptoms of depression in participants categorised as MCI were significantly greater (OR = 1.71, CI = 1.10-2.67 *p* = .018) than for those participants categorised as NCI.

### The association between MCI and social networks

A multiple regression analysis which controlled for age, sex, and number of health conditions revealed that there was a significant difference in social networks between participants with MCI and those categorised as NCI, *t* = 3.48, *p* = .001. Participants in the MCI category scored lower than those in the NCI category, with a mean difference of 1.51 points on the LSNS6, indicating that they had weaker social networks.

### The association between social networks and mood

Logistic regression analyses were conducted to investigate the association between social network score and mood. The analyses showed that social network score was associated with slightly lower odds of having symptoms of anxiety (OR = 0.93, CI = 0.90-0.97, *p* < .001) and depression (OR = 0.94, CI = 0.92-0.97, *p* < .001); as social network score increases, the odds of experiencing symptoms of anxiety and depression are lower. Table 2 shows the mean social network scores for each AGECAT level of anxiety and depression and suggests a general trend whereby social network scores are lower as severity of anxiety and depression increase.

**Table 2:** Mean social network score for each AGECAT level of anxiety and depression

Anxiety	N	Mean Social Network Score (SD)	Depression	N	Mean Social Network Score (SD)
0	1646	15.87 (5.74)	0	1982	15.90 (5.70)
1	1018	15.22 (5.82)	1	136	14.46 (6.42)
2	51	15.47 (5.72)	2	460	15.23 (5.73)
3	69	13.72 (6.23)	3	168	14.11 (6.21)
4	24	13.96 (6.20)	4	67	13.33 (5.52)
5	5	12.20 (6.61)			

### Do social networks mediate the relationship between MCI and mood?

**Fig. 1:**
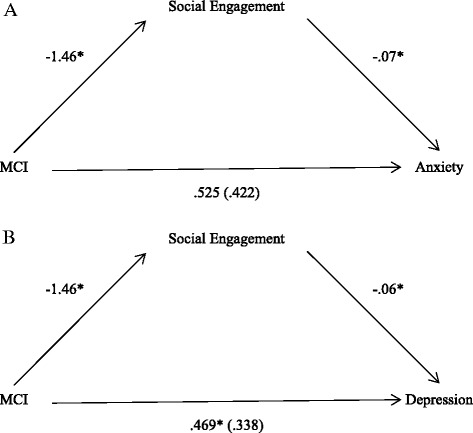
Social network score mediates the relationship between mood and MCI. Panel **a** shows the mediation of the relationship between anxiety and MCI by social network score, and panel **b** shows the mediation of the relationship between depression and MCI by social network score. Significant coefficients (p=.05) are denoted by the * symbol. The coefficients generated for the relationship between mood and MCI when social network score is included are shown in parentheses

Mediation was examined using logistical regression analyses and the four step method advised by Baron and Kenny (1986) [24]. In the first step, the relationship between mood and MCI was tested by regressing mood upon MCI to investigate the odds of experiencing mood problems for participants with MCI and without. Next, the relationship between social network score and MCI was tested in the second step by regressing social network score upon MCI. Lastly the relationship between mood and MCI, with social network score as a covariate, was tested using regression. Age, gender and the total number of health conditions were controlled for during all steps. Full mediation can be demonstrated when the effect of mood on MCI is no longer significant in the presence of social network score, and the significance of the mediation effect can be confirmed using Sobel’s test. Our analyses suggest that social networks mediate the relationship between MCI and anxiety (Fig. 1, panel a), and this relationship is significant according to Sobel’s test (z’ = 2.55, *p* = .010). Social networks mediate the relationship between MCI and depression (Fig. 1, panel b), and again this relationship is significant according to Sobel’s test (z’ = 2.83, *p* = .004). The two subscales of the LSNS were further examined independently to investigate whether it is engagement with either friends or relatives that drives the mediation of the relationship between MCI and mood problems. For both anxiety and depression, each subscale could only partially mediate the relationship, highlighting that full mediation may only be achieved when both friends and relatives are considered in the analysis.

## Discussion

Our findings clearly show that social networks are involved in the relationship between MCI and mood problems, and can account for the variance in this relationship. People with MCI tend to be have less rich social networks than those without cognitive impairment, and having weaker social networks is associated with a higher likelihood of having anxiety or depression. Including both relatives and friends in the social network are important in mediating the association between MCI and mood problems which is interesting to note when developing interventions to boost social networks. These findings add to previous literature by drawing from a large sample that is representative of a range of living situations, and we believe that this is the first time that mediation analyses have been used to explore the relationship between MCI and mood in this way.

These findings are in line with previous literature which suggests that social integration can lead to better mental health outcomes [10], prevent depression [9], and decrease psychological distress [6], whereas social isolation can increase the risk of depression [4]. Social support may be galvanised into action during times of stress, which can mitigate any negative effects of potential stressors and help to improve well-being [15], and may foster health promoting environments or behaviours through the provision of advice, assistance, or access to resources [25]. Social networks can provide opportunities for cognitive stimulation, and can provide a dynamic environment in which to utilise various cognitive functions [26]. Whilst longitudinal research does not favour the reverse causation hypothesis, which suggests that cognitive decline reduces ability to maintain social networks and manage interactions, [16], cognitive decline may lead to a weakening of social networks. The mechanism through which the relationship between mood, social networks, and MCI operates could be that having stronger social networks enables people to draw on support (both emotional and practical), and cultivate a sense of purpose which can lead to a positive self-image that operates as a buffer in stressful times or anxiety provoking situations. This process may then help to reduce mood problems or ameliorate their harmful effects on cognitive functioning, and prevent or delay cognitive decline. However, the opposite may also be true in that cognitive decline may make it more difficult for people to participate in social situations, leading to weaker social networks, which in turn may lead to feelings of depression or anxiety. It is not possible to investigate directionality in this study because it is cross-sectional in design, but further research using longitudinal data may establish this.

The present study does have some methodological limitations. Firstly, the study is cross-sectional in design, which does not allow for directionality to be explored or established. Additionally, there are small numbers of people reporting anxiety, which may be due to underreporting as older people may attempt to minimise anxious feelings or worries. The AGECAT algorithm [20] used to calculate which participants had anxiety was developed 30 years ago, and consequently may not accurately capture anxious feelings or worries that are pertinent to older people today.

However, there are many strengths of this study. The overall sample used in the analyses is large and draws from both urban and rural populations. The sampling frame was designed to over-sample for people over the age of 75 to ensure that the oldest old were well represented within the sample, and the participation rate was almost 50%. Additionally, the Lubben Social Network Scale six-item version (LSNS-6) [22] was used as the measure of social networks in this study and is a reliable and valid measure. The LSNS-6 was found to have good internal consistency (α = 0.83) and discriminant validity, of the whole measure and for both subscales [22], and showed good internal consistency when used in this study (α = 0.73).

The findings from this study have several potential applications. Firstly, the findings suggest that attempts to support, strengthen and extend social networks may be beneficial. However, there can be practical barriers that stop older people participating socially, such as lack of adequate transport or convenient places to meet others, and so many reasons why older people do not engage socially are beyond their control [27]. Organisations, local government and charities could be encouraged to facilitate opportunities for social participation, such as providing a space for people to meet or increasing the availability of transport for older people who cannot drive or live in rural areas. Studies involving programmes to enrich friendship [28] and prevent social isolation [29] are effective at increasing participants’ number of friendships and reducing loneliness.

## Conclusions

This study aimed to investigate how social networks may be involved in the relationship between MCI and mood and found that weaker social networks are associated with both mood problems and MCI. Our results showed that social networks do mediate the relationship between MCI and mood, and consequently interventions that target social participation may have beneficial effects in reducing both cognitive decline and mood problems in older people.

## Abbreviations

CAMCOG: Cambridge Cognitive Examination
CFAS: Cognitive Function and Ageing Study
LSNS-6: Lubben Social Network Scale six-item version
MCI: Mild cognitive impairment
NCI: No cognitive impairment
